# 

**Published:** 1885-09

**Authors:** 


☞THE PROMPT REMITTANCE OF SUBSCRIPTIONS is absolutely necessary to enable us to maintain this work, so acceptably begun, and so full of promise.☜

☞ Gentlemen, please remit your Subscriptions as early as possible. It is of vital importance to the Journal. Don't postpone it. ☜

